# Implant-Grade Neural Sensing with Artifact-Resilient Computational Intelligence Preserves Spatial Fidelity of HFO Biomarkers in Epilepsy

**DOI:** 10.21203/rs.3.rs-10657493/v1

**Published:** 2026-08-24

**Authors:** Behrang Fazli Besheli, Amir Hossein Ayyoubi, Chandra Prakash Swamy, Jhan Luke Okkabaz, Jamie J. Van Gompel, Kai J. Miller, W. Richard Marsh, Nicholas M. Gregg, Gregory A. Worrell, Nuri F. Ince

**Affiliations:** 1. Department of Neurologic Surgery, Mayo Clinic, Rochester, MN, USA.; 2. Department of Bioinformatics and Computational Biology, University of Minnesota, Minneapolis, MN, USA.; 3. Department of Neurology, Mayo Clinic, Rochester, MN, USA.; 4. Department of Physiology and Biomedical Engineering, Mayo Clinic, Rochester, MN, USA.

## Abstract

High-frequency oscillations (HFOs) are clinically established biomarkers of epileptogenic tissue, yet their translation from hospital-based recording systems to implantable neural interfaces remains a major barrier to their utilization in adaptive neuromodulation in epilepsy. Here, we established a bedside translational framework using a benchtop implementation of a wireless implantable neural interface (Brain Interchange, BIC) to evaluate whether clinically meaningful HFO can be preserved under implant-grade sensing constraints. Ten patients with drug resistant epilepsy underwent simultaneous 24-hour intracranial EEG recordings using synchronized BICs and a clinical-grade amplifier. A sensitive detector identified candidate HFOs, while sparse signal processing and machine learning methods removed artifact related pseudo-HFOs. Despite its lower sampling rate, narrower analog bandwidth, and higher noise floor, the integrated sensing and computational intelligence framework preserved approximately 82% of clinical amplifier HFOs and maintained nearly identical spatial distribution of pathological activity. After pseudo-HFO elimination, BIC derived HFOs localized the seizure onset zone with 84% sensitivity and 90% specificity, comparable to the clinical amplifier despite lower fast ripple detection. These findings demonstrate that implant-grade neural sensing, when integrated with artifact resilient computational intelligence, can preserve clinically relevant HFO biomarkers and their spatial fidelity without reproducing every individual electrophysiological event. More broadly, this work establishes a translational framework for chronic tracking of evolving epileptogenic networks and future biomarker-guided adaptive neuromodulation.

## Introduction:

The clinical success of neuromodulation across a broad spectrum of neurological and psychiatric disorders underscores its therapeutic potential for modulating dysfunctional brain networks^1-3^. Historically, neuromodulation therapies were delivered in an open-loop^4^ or closed-loop^5^ manners, relying on fixed stimulation parameters that did not adjust to ongoing changes in neural activity or patient state. Recent progress, however, has enabled dynamic, adaptive paradigms capable of adjusting therapy in real time to evolving brain states^6-14^.

This technological evolution has been driven by the emergence of sensing-enabled and bidirectional neuromodulation platforms. Systems such as NeuroPace responsive neurostimulation, which continuously senses electrocorticographic activity and delivers stimulation in response to detected neural patterns, illustrate the clinical utility of closed-loop non-adaptive strategies in epilepsy^15,16^. In parallel, sensing-enabled deep brain stimulation devices, including the Medtronic Percept platform^17-19^ and its investigational predecessor the Summit RC+S^20-24^, have demonstrated the value of capturing chronic local field potentials alongside therapeutic stimulation to inform patient-specific adjustments. While these systems highlight the clinical importance of neural sensing, most remain constrained in bandwidth and sampling rate, limiting their ability to capture high-frequency biomarkers that may be critical for precise seizure onset zone (SOZ) localization.

A central requirement for adaptive neuromodulation is the ability to sense neural activity with sufficient temporal resolution and signal quality to identify pathologic neurobiomarkers. In epilepsy, high-frequency oscillations (HFOs), including ripples (R, 80–200 Hz) and fast ripples (FR, 200–500 Hz), have emerged as well-established biomarkers of epileptogenic tissue and seizure-generating networks^25-30^. Despite their clinical value, translation of HFOs into implantable systems has been limited because most devices lack the bandwidth and sampling rate needed to reliably capture these high-frequency events^31^. As a result, the potential of HFO-based closed-loop stimulation remains largely unrealized.

To address this gap, we combined this implantable platform with a machine-learning–based artifact-rejection pipeline to evaluate the feasibility of HFO detection in ten patients with drug-resistant epilepsy undergoing invasive monitoring, using a benchtop version of a next-generation wireless neural interface (Brain Interchange System, BIC; CorTec). Intracranial EEG (iEEG) signals were split between the BIC and an FDA-approved clinical amplifier (CA), enabling sample-accurate alignment and unbiased comparison over 24 hours in the epilepsy monitoring unit (EMU). This framework allowed us to benchmark event detection, spectral characteristics, and noise properties across both systems. By quantifying how well the implantable platform captures clinically meaningful HFOs relative to a clinical gold standard, we provide a rigorous translational assessment of its suitability for future HFO-driven closed-loop neuromodulation. These results establish a quantitative foundation for next-generation implantable systems designed to sense epileptogenic high-frequency activity and support biomarker-guided adaptive therapy.

## Results

**Fig. 1:**
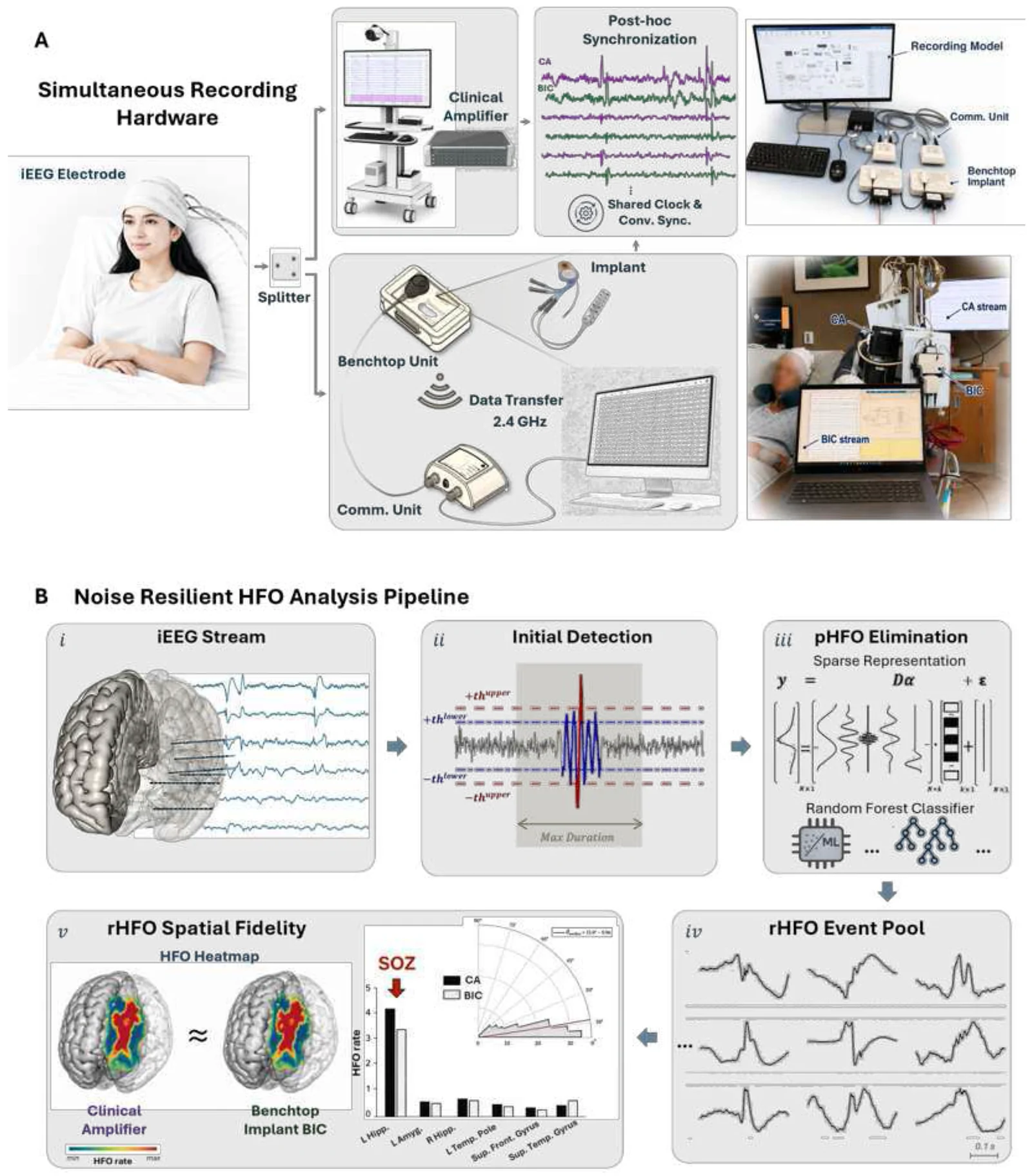
Simultaneous recording hardware and noise-resilient HFO analysis pipeline. **(A)** Simultaneous recording hardware used to acquire parallel clinical amplifier and BIC data streams during EMU monitoring. Signals from selected iEEG contacts were split and routed simultaneously to the clinical Natus Quantum amplifier and to the investigational BIC system. The schematic shows the clinical amplifier stream, post hoc synchronization, and the BIC pathway, including the benchtop unit, implant interface, 2.4 GHz data transfer, communication unit, and recording computer. For visualization clarity, the schematic illustrates a single BIC pathway, whereas the study used a dual-BIC configuration with two independent BIC units operated in parallel. In this configuration, 64 contacts, 32 per unit, were selected for broad spatial coverage. Representative photographs show the recording model, BIC communication and benchtop units, and EMU deployment during concurrent clinical amplifier and BIC recording. **(B)** Noise-resilient HFO analysis pipeline applied to temporally aligned clinical amplifier and BIC recordings. The workflow included synchronized iEEG streams, initial HFO candidate detection, pseudo-HFO elimination using sparse signal representation and machine learning classification, and generation of a real HFO event pool. Spatial fidelity was evaluated by comparing BIC and clinical amplifier HFO rate maps and regional HFO distributions relative to the epileptogenic zone.

Figure 1 provides an overview of the experimental and analytical framework used in this study. The simultaneous recording configuration used to acquire intracranial signals with the BIC and clinical amplifier is shown in Fig. 1A, while the noise-resilient analysis pipeline developed for HFO detection, artifact elimination, and assessment of spatial biomarker fidelity is summarized in Fig. 1B.

### Spectral and Noise Characteristics of BIC and Clinical Recordings

**Fig. 2:**
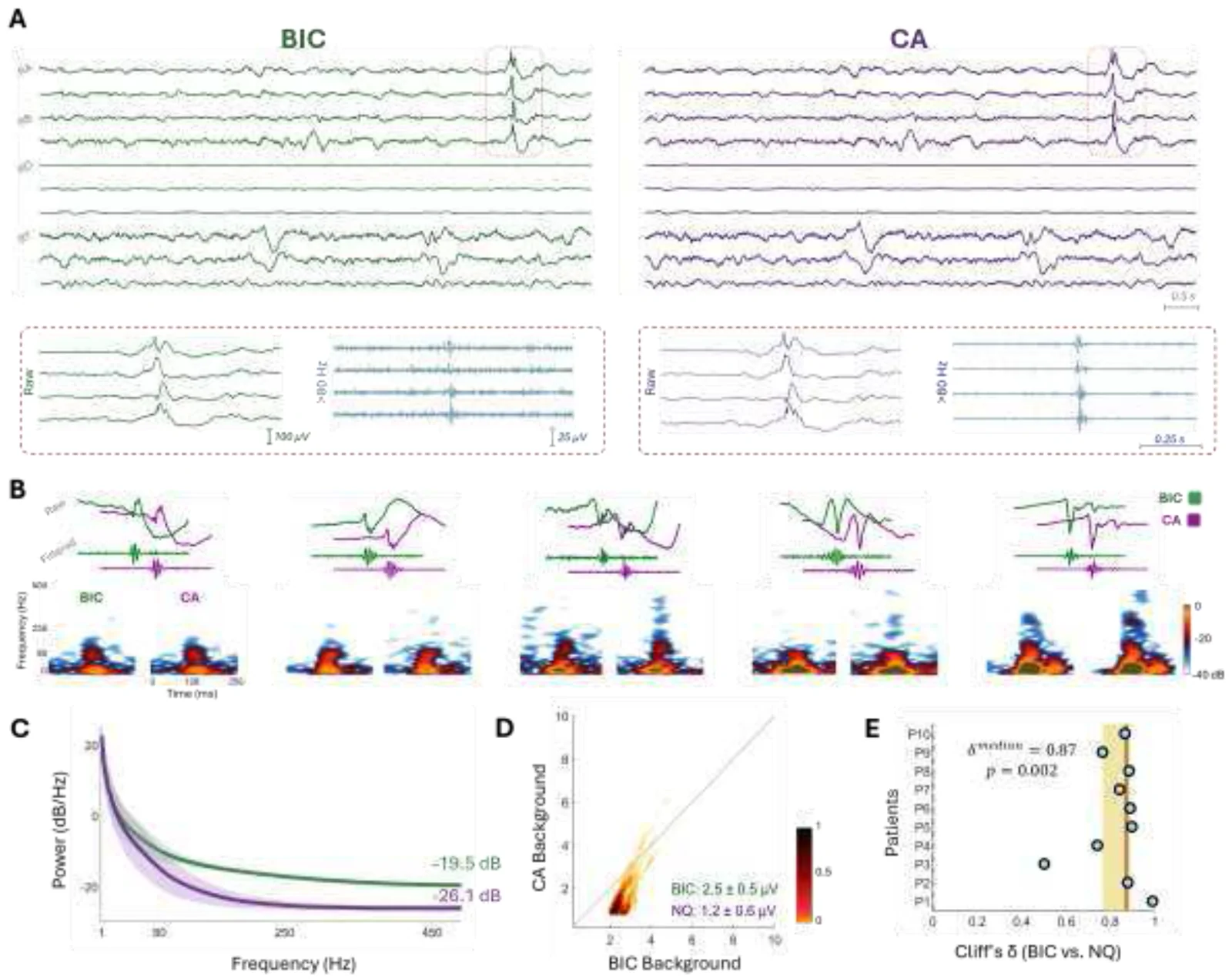
Comparison of signal characteristics and background noise between BIC and clinical recordings. **(A)** Representative 10-s segments from 10 iEEG channels recorded simultaneously by the BIC and corresponding clinical amplifier (CA) systems. Raw iEEG traces are shown with BIC signals in green and CA signals in purple. A magnified 1-s segment containing an HFO event is shown for each system to illustrate waveform morphology and high-frequency oscillatory content. **(B)** Representative HFO events detected in the BIC system (green) and their time-matched corresponding events recorded by the clinical CA system (purple). For each event, the raw iEEG waveform (top), the HFO-band–filtered signal (80–500 Hz; bottom), and the time–frequency representation computed using the short-time Fourier transform are shown. **(C)** Average power spectral density (PSD) computed across all non-corrupted channels and subjects. Both systems exhibit comparable spectral characteristics at low frequencies (0–60 Hz). At higher frequencies (>200 Hz), BIC recordings show a higher noise floor, plateauing near −19 dB, whereas CA recordings exhibit a lower noise floor, approximately −26 dB. **(D)** Paired density plot comparing HFO-band (80–500 Hz) root-mean-square (RMS) values between BIC and CA across all channels, subjects, and non-overlapping 10-min segments over 24 hours. Each point represents a single 10-min segment. RMS values are more densely clustered around ~2.5 μV for BIC and ~1.2 μV for CA, reflecting differences in amplifier noise characteristics. **(E)** Subject-level Cliff’s delta (δ) quantifying the degree to which HFO-band RMS values were higher in BIC than in CA recordings. For each subject, δ was computed from the distributions of RMS values across all included recording segments. Positive values indicate higher RMS values in BIC. Points represent subject-level δ values, the solid vertical line marks the group median, and the shaded region indicates the 95% bootstrap confidence interval of the median across subjects. BIC recordings exhibited consistently higher HFO-band RMS values across participants (median δ = 0.871, 95% CI, 0.764 to 0.890; Wilcoxon signed-rank test against zero, *p* = 0.002).

To provide a representative example of signal quality, raw iEEG traces from 10 channels recorded simultaneously by the BIC and CA systems are shown in Fig. 2A. BIC signals are shown in green and CA signals in purple. A 1-s segment is enlarged for each system to highlight both low-frequency waveform morphology and high-frequency activity. At low frequencies, the waveforms are nearly identical between systems, whereas in the HFO band the BIC recording exhibits a thicker high-frequency background compared with CA. A complementary example illustrating signal characteristics in the high-frequency range is provided in Supplementary Fig. S2, which shows raw and HFO-band–filtered iEEG from a hippocampal SOZ channel together with short-time Fourier transform (STFT) representations for both systems. In this example, the BIC recording exhibits increased background activity in the FR band compared with CA, which may mask FR events in the time–frequency representation.

Representative examples of detected HFOs in BIC recordings and their time-matched counterparts in the CA recordings are shown in Fig. 2B. For each event, the raw iEEG waveform, the HFO-band–filtered signal, and the corresponding STFT are displayed to illustrate time–frequency characteristics. For events with dominant frequency components below approximately 200 Hz, both BIC and CA recordings capture oscillatory activity clearly and consistently. In contrast, for higher-frequency HFOs, including fast ripples with dominant components above approximately 200 Hz, fast activity may be less prominent or not fully visible in the BIC time–frequency representations due to elevated high-frequency noise levels. Importantly, these events typically retain oscillatory components within the ripple band, which remain detectable in the BIC recordings. Additional multi-channel examples are provided in Supplementary Figs. S3 and S4, which illustrate HFO detection across simultaneous channels for BIC and CA recordings, respectively. In the BIC example (Supplementary Fig. S3), fast HFO activity is less clearly distinguishable from the elevated high-frequency background in the HFO-filtered signals, which may contribute to fewer detected events across channels. In contrast, the corresponding CA example (Supplementary Fig. S4) shows clearer isolation of HFO activity with minimal masking from background noise, enabling more consistent detection across channels. Together, these examples indicate that increased high-frequency background activity in BIC recordings may limit the detection of fast HFO events relative to CA.

The average power spectral density (PSD) across all non-corrupted channels and subjects is shown in Fig. 2C. Both recording systems exhibit comparable spectral characteristics at low frequencies (0–60 Hz). At higher frequencies (>200 Hz), differences in noise characteristics become apparent: BIC recordings exhibit a higher noise floor, plateauing near −19 dB, whereas CA recordings maintain a lower noise floor of approximately −26 dB.

To further quantify background activity in the HFO band, root-mean-square (RMS) values computed across channels, subjects, and non-overlapping 10-minute segments are summarized in Fig. 2D using a paired density plot. Each point compares time-matched BIC and CA RMS values from a single 10-minute segment. The highest-density regions were centered near 2.5 μV for BIC and 1.2 μV for CA, reflecting the different background-noise characteristics of the two recording systems. Across all segments and subjects, HFO-band RMS values were significantly higher in BIC than in CA recordings (*p < 0.001).*

Figure 2E summarizes the difference at the subject level using Cliff’s delta (δ) to quantify the degree to which HFO-band RMS values were higher in BIC than in CA recordings. For each subject, Cliff’s delta was computed from the distributions of RMS values across all included recording segments, with positive values indicating higher RMS values in BIC. Points denote subject-level δ values, the solid vertical line marks the group median, and the shaded region represents the 95% bootstrap confidence interval of the median across subjects. BIC recordings exhibited consistently higher HFO-band RMS values across subjects (median δ = 0.871, 95% CI, 0.764 to 0.890; Wilcoxon signed-rank test against zero, *p* = 0.002), indicating that the difference was not driven by a small subset of participants.

### Wireless Transmission Stability During Prolonged Recording

**Fig. 3:**
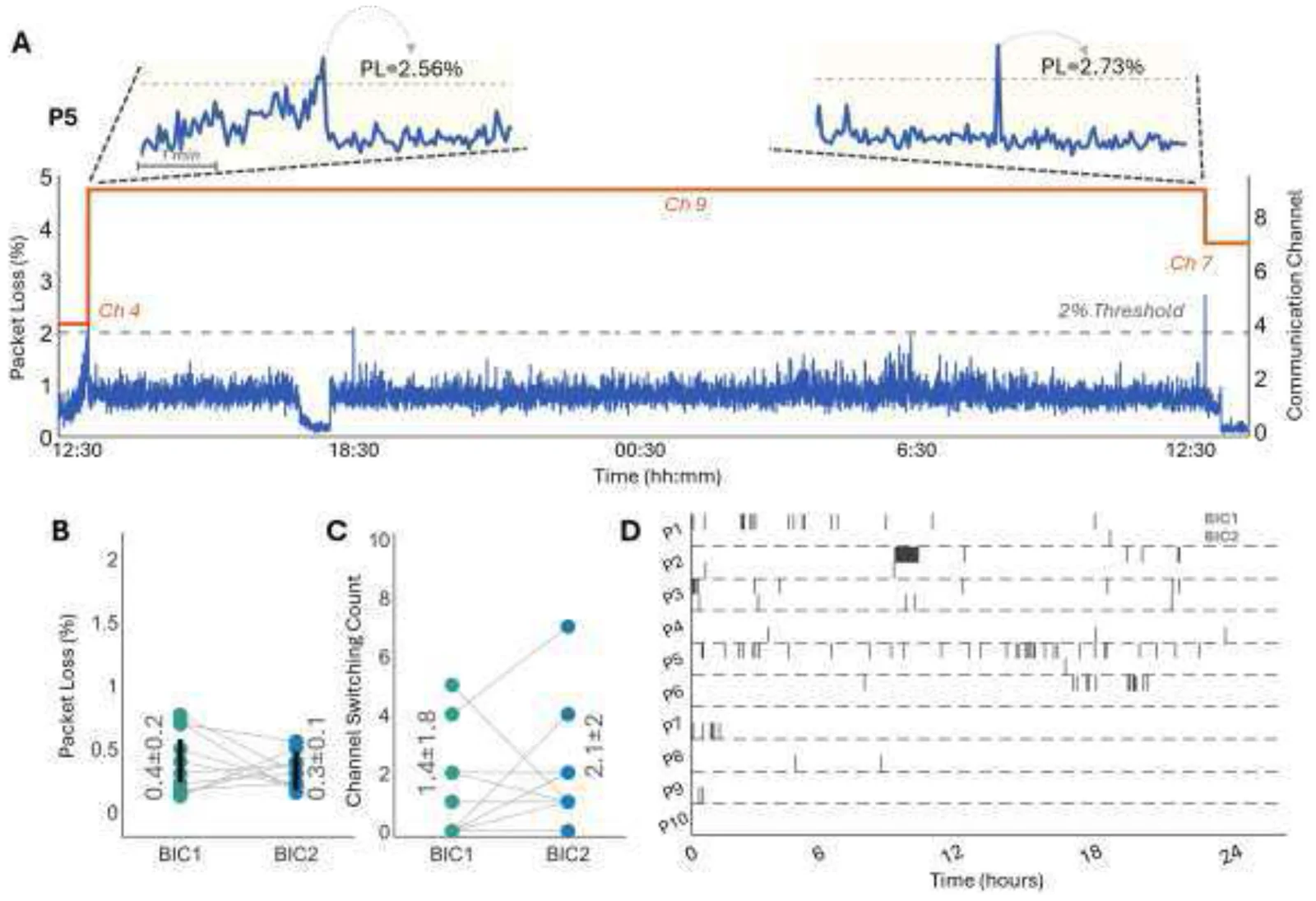
Packet loss and communication channel stability during prolonged BIC recordings. **(A)** Packet loss over 24 hours from a representative BIC recording. Packet loss percentage for BIC1 is shown in blue, and the active communication channel is shown in orange. Two channel switching events occurred during the recording. Channel switching was triggered when packet loss exceeded 2 percent for longer than 10 seconds. Insets show a 5-minute zoom around each switching event, illustrating transitions from channel 4 to 9 when packet loss reached 2.56 percent, and from channel 9 back to 4 when packet loss reached 2.73 percent. **(B)** Summary of packet loss across all subjects for BIC1 and BIC2. Mean packet loss over the full recording duration remained below 1 percent for both devices, with 0.4 ± 0.2 percent for BIC1 and 0.3 ± 0.1 percent for BIC2. **(C)** Number of communication channel switches during 24-hour recordings for each subject. Channel switching counts were low for both devices, with 1.4 ± 1.8 switches for BIC1 and 2.0 ± 1.2 switches for BIC2, with no significant difference between devices across subjects (p = 0.5). **(D)** Temporal distribution of packet loss over 24 hours across all subjects for BIC1 and BIC2. White regions indicate time periods with packet loss below 1 percent, while gray regions denote periods exceeding 1 percent packet loss

Wireless data in the BIC system are transmitted from the implant to an external communication unit, during which a fraction of data packets may be lost. Packet loss (PL) quantifies the proportion of missing samples introduced during this wireless transfer, and communication channel switching reflects changes between predefined radio frequency channels used to maintain stable transmission. To assess the reliability of wireless data transfer during prolonged recordings, PL and channel behavior were analyzed over continuous 24-hour recordings (Fig. 3). Across all subjects and both implantable devices, PL remained low throughout 24-hour recordings. Mean packet loss was below 1% for both BIC1 and BIC2, and more than 98% of the total recording time was characterized by packet loss below this threshold (Fig. 3B, D). Communication channel switching events were infrequent, with an average of fewer than two switches per 24 hours for both devices and no significant difference between BIC1 and BIC2 (*p=0.5*, Fig. 3C). More information on communication channels and device specifications for the BIC system is provided in Supplementary Table 1. Channel switching events occurred, based on a predefined threshold applied to both BICs, only when PL transiently exceeded 2% for longer than 10 seconds, as illustrated in a representative example in Fig. 3A. These incidents were isolated and followed by rapid stabilization of PL after switching (Fig. 3D).

### Pseudo-HFO Classification Across Recording Systems

**Fig. 4:**
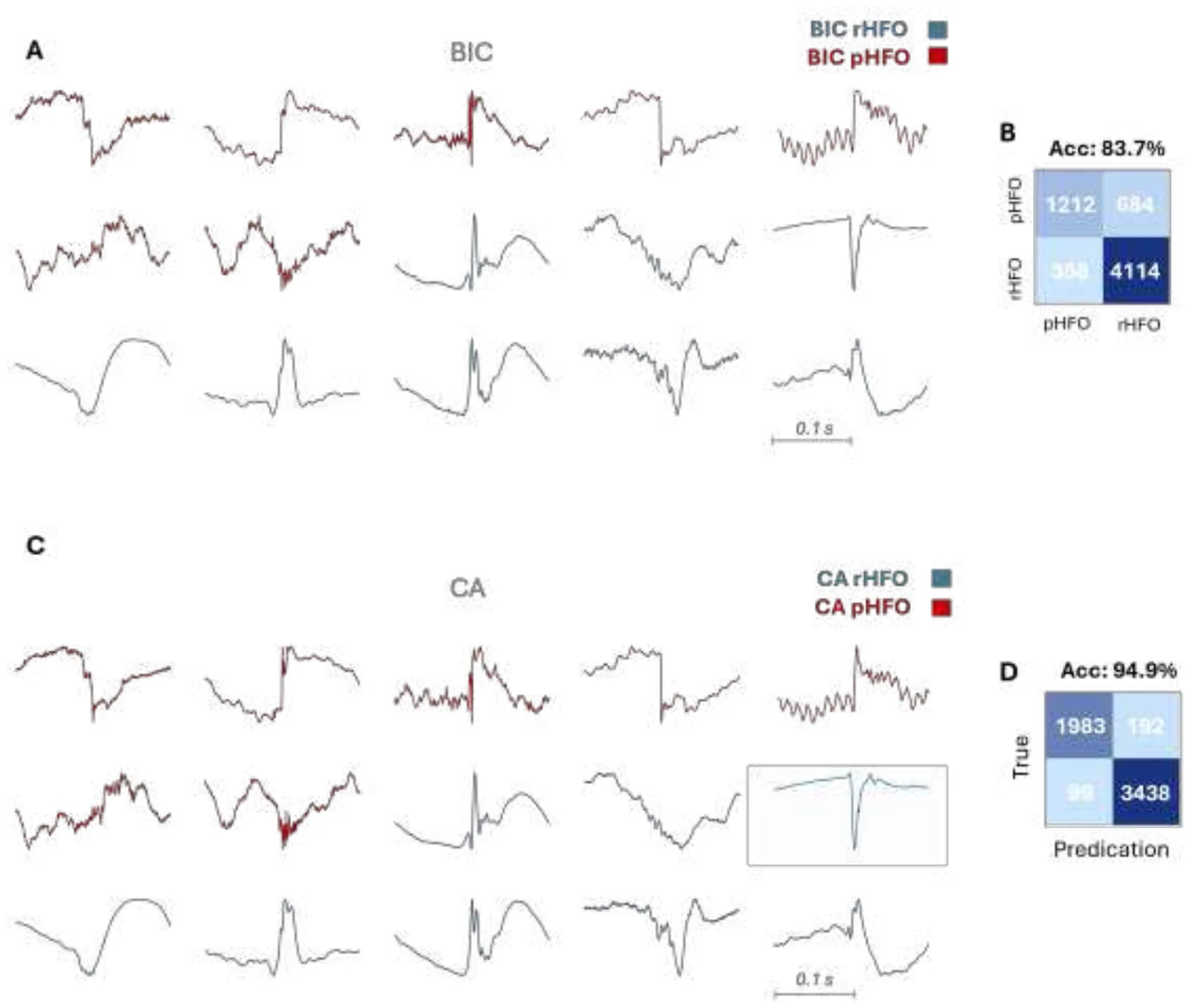
Pseudo-HFO characterization and classification performance in implantable and clinical recordings. **(A)** Representative examples of real HFOs (black) and artifact-related pseudo-HFOs (red) detected in Brain Interchange System (BIC) recordings following initial amplitude-based HFO detection. Event labels were assigned by expert visual review. **(B)** Confusion matrix summarizing pseudo-HFO classification performance for labeled BIC events using the sparse representation–based RF-OMP (random forest – orthogonal matching pursuit) classifier. **(C)** Corresponding examples of real HFOs (black) and pseudo-HFOs (red) detected in clinical amplifier recordings (Natus Quantum, CA) after identical initial detection, with expert-assigned labels. **(D)** Confusion matrix showing classification performance for labeled CA events using the same RF-OMP pseudo-HFO elimination pipeline.

The original pseudo-HFO (pHFO) classification model was trained on iEEG sampled at 2 kHz, preserving spectral content up to approximately 1 kHz. In contrast, BIC recordings were acquired at 1 kHz, corresponding to a Nyquist frequency of approximately 500 Hz and a lower effective analog bandwidth at 325 Hz. This reduced bandwidth alters the spectral representation of high-frequency transients, particularly sharp, non-neural deflections. As a result, delta-shaped artifacts that commonly generate pHFO detections appear temporally smoother in BIC recordings than in CA recordings, making discrimination between real HFOs and artifacts more challenging (Fig. 4A, C).

To explicitly evaluate the impact of reduced bandwidth on pHFO classification, a subset of events (~500 events per subject) from both recording systems was independently labeled as real HFOs or pHFOs by three expert reviewers using majority voting. BIC events were linearly upsampled to 2 kHz before feature extraction to satisfy the input requirements of the original model architecture. Classification performance was evaluated on more than 10,000 labeled events from BIC and from CA recordings. Representative examples from both systems illustrate the smoother temporal profiles of pHFOs in BIC compared to CA, as shown in Fig. 4.

Quantitatively, the pHFO classifier achieved an accuracy of approximately 84% for BIC labeled events, compared to ~94% for CA events (Fig. 4B, D). Although classification performance was reduced for BIC recordings, accuracy remained sufficient for reliable artifact elimination. Accordingly, the same pHFO elimination pipeline was applied to both BIC and CA datasets prior to all downstream analyses.

### HFO Rates and Cross-System Spatial Alignment

**Fig. 5:**
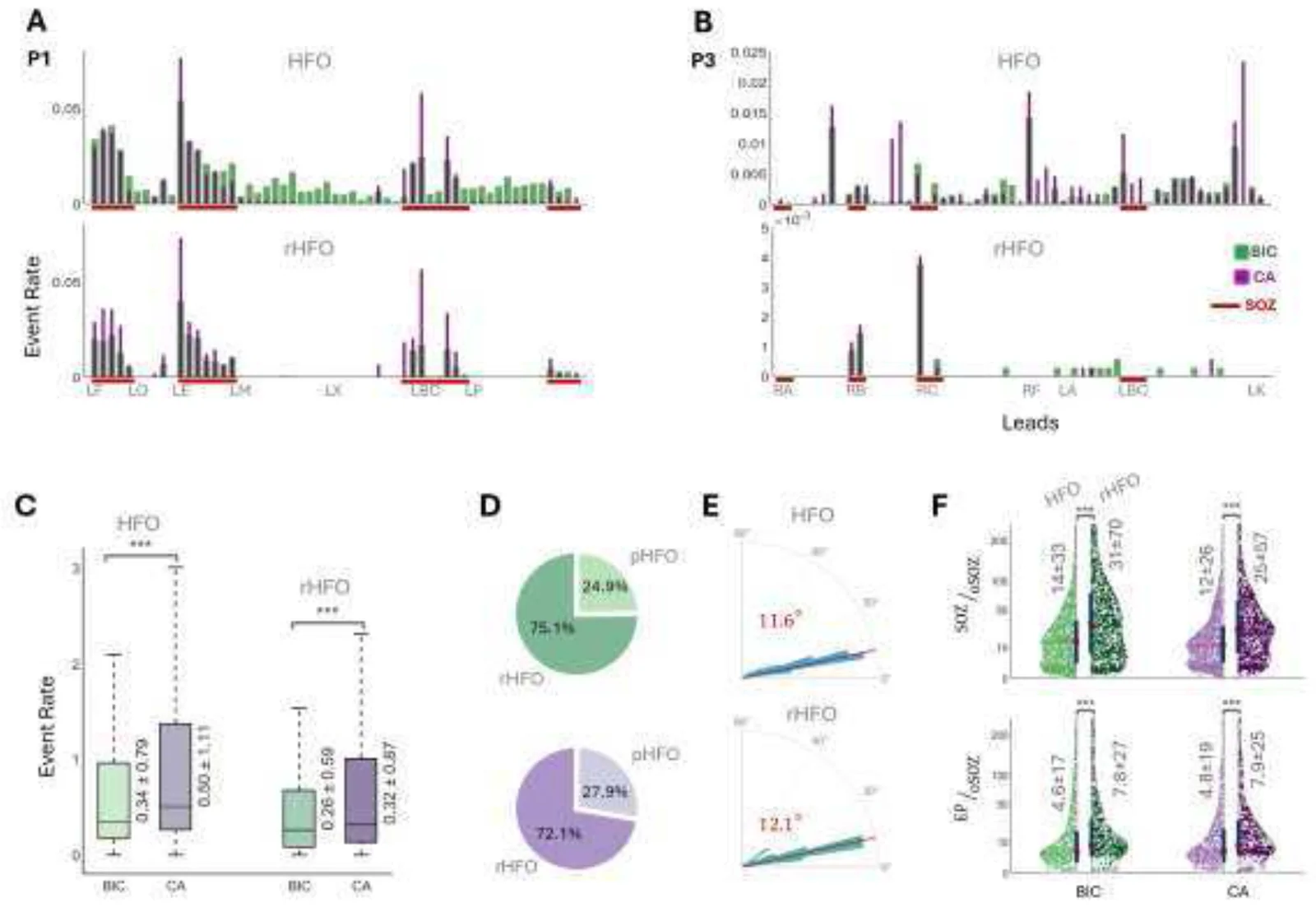
Effect of pseudo-HFO elimination on spatial distribution, event rates, and SOZ specificity. **(A, B)** Spatial distribution of channel-wise HFO event rates for a 1-hour recording segment from representative patients P1 (A) and P3 (B). For each patient, distributions are shown before pseudo-HFO elimination (top) and after denoising (bottom), with BIC shown in green and CA shown in purple. Channels clinically identified as belonging to the seizure onset zone (SOZ) are indicated by a red line beneath each distribution. Pseudo-HFO elimination reduces diffuse background detections, yielding sparser spatial distributions that more closely align with clinically defined SOZ channels. **(C)** Boxplots of HFO event rates across all subjects and non-overlapping 10-minute segments for BIC and CA recordings, shown before and after pseudo-HFO elimination. Distributions include all detected HFOs (HFO and rHFO). Across conditions, CA captures significantly higher event rates than BIC both before and after denoising. Following pseudo-HFO elimination, approximately 82% of CA-detected events are also captured by BIC. **(D)** Proportion of candidate HFOs classified as rHFOs and pHFOs for BIC and CA recordings across all subjects. Overall, 24.9% of candidate HFOs in BIC and 27.9% in CA were identified as pHFOs. **(E)** Spatial alignment of channel-wise HFO distributions between BIC and CA recordings across all subjects over the full 24-hour recording duration. Each data point represents one non-overlapping 10-minute multichannel segment comparing BIC with time-matched CA data. Angular differences derived from cosine similarity are shown for initial candidate HFOs (blue) and for rHFOs after pseudo-HFO elimination (green). Median angular differences were 11.6° before denoising and 12.1° after denoising, indicating preserved spatial concordance following artifact rejection. **(F)** Violin plots showing the proportion of events originating from SOZ channels relative to non-SOZ channels (SOZ/oSOZ) and the epileptogenicity ratio (EP/oSOZ) for BIC and CA recordings, before and after pseudo-HFO elimination. For both recording systems, pseudo-HFO elimination resulted in a significant increase in SOZ specificity and EP contrast (*p < 0.001*).

Representative examples of channel-wise HFO spatial distributions from 1-hour recording segments in two patients (P1 and P3) are shown in Fig. 5A, B. For both patients, initial HFO candidates were diffusely distributed across channels, with elevated rates extending beyond clinically defined SOZ contacts. Following pHFO elimination, the spatial distributions became markedly sparser, with HFO activity concentrating around SOZ channels. This effect was consistently observed in both BIC and CA recordings, indicating that denoising preferentially removed broadly distributed detected high-frequency artifacts while preserving focal real-HFOs aligned with clinically annotated seizure-generating regions.

HFO rates were quantified for initial candidate events (HFO), remaining real-HFOs (rHFO; Fig. 5C). Initial HFO rates were higher in CA than in BIC (BIC: 0.34 ± 0.79; CA: 0.50 ± 1.11; BIC/CA - ratio = 0.68). After pseudo-HFO elimination, rHFO rates remained higher in CA (BIC: 0.26 ± 0.59; CA: 0.32 ± 0.87), but the BIC/CA ratio increased to 0.82. These results indicate that although CA consistently captures a greater number of HFO events, BIC retains more than 80% of CA-detected HFOs after pHFO elimination.

The overall proportion of candidate HFOs classified as pHFOs is summarized in Fig. 5D. Across all subjects, 24.9% of candidate HFOs detected in BIC recordings and 27.9% in CA recordings were identified as pHFOs, indicating comparable levels of artifact rejection across systems despite differences in baseline noise characteristics.

Cross-system spatial consistency was evaluated using non-overlapping, synchronized 10-minute segments from all subjects. Cosine similarity was computed between the channel-wise HFO distributions derived from BIC and CA recordings before and after pHFO elimination. Before pHFO elimination, spatial alignment of all candidate events exhibited a median angular difference of 11.6° (cosine similarity ≈ 0.979). After pHFO elimination, spatial alignment remained highly consistent, with a marginal change to 12.1° (cosine similarity ≈ 0.977), indicating that pseudo-HFO elimination did not disrupt the underlying spatial concordance between the two systems (Fig. 5E).

Regional specificity was further assessed by calculating the ratio of event rates in SOZ channels to those in outside-SOZ channels (SOZ/oSOZ), as well as the ratio of event rates in early-propagation channels to those in outside-SOZ channels (EP/oSOZ), for both BIC and CA recordings (Fig. 5F). In both systems, pHFO elimination significantly increased the SOZ-to-oSOZ and EP-to-oSOZ contrasts (*p < 0.001*). The SOZ/oSOZ ratio increased from 14 to 31 for BIC and from 12 to 25 for CA following pHFO elimination. Similarly, the EP/oSOZ ratio increased from 4.6 to 7.8 for BIC and from 4.8 to 7.9 for CA. Normalized HFO rates across anatomical regions are shown in Supplementary Fig. S5.

### Epileptogenic Network Localization Using BIC- and clinical-Derived HFOs

To assess SOZ localization, we quantified the spatial distribution of HFOs across all non-overlapping 10-minute segments and all subjects using an SOZ likelihood metric. Prior to artifact removal, accurate SOZ localization failed in three subjects (P3, P9, and P10) for both BIC and CA recordings, reflecting contamination by non-neuronal high-frequency activity. Following pseudo-HFO elimination, localization performance improved substantially, with only one remaining failure case (P9). P9 represented a clinically inconclusive SOZ case: although candidate SOZ contacts were identified during prolonged EMU monitoring, a confident focal epileptogenic network could not be delineated, and the patient ultimately received deep brain stimulation rather than focal resection or ablation. This concordance between HFO-based localization failure and clinical uncertainty supports the biological relevance of the observed results rather than indicating a limitation of the recording system.

**Fig. 6:**
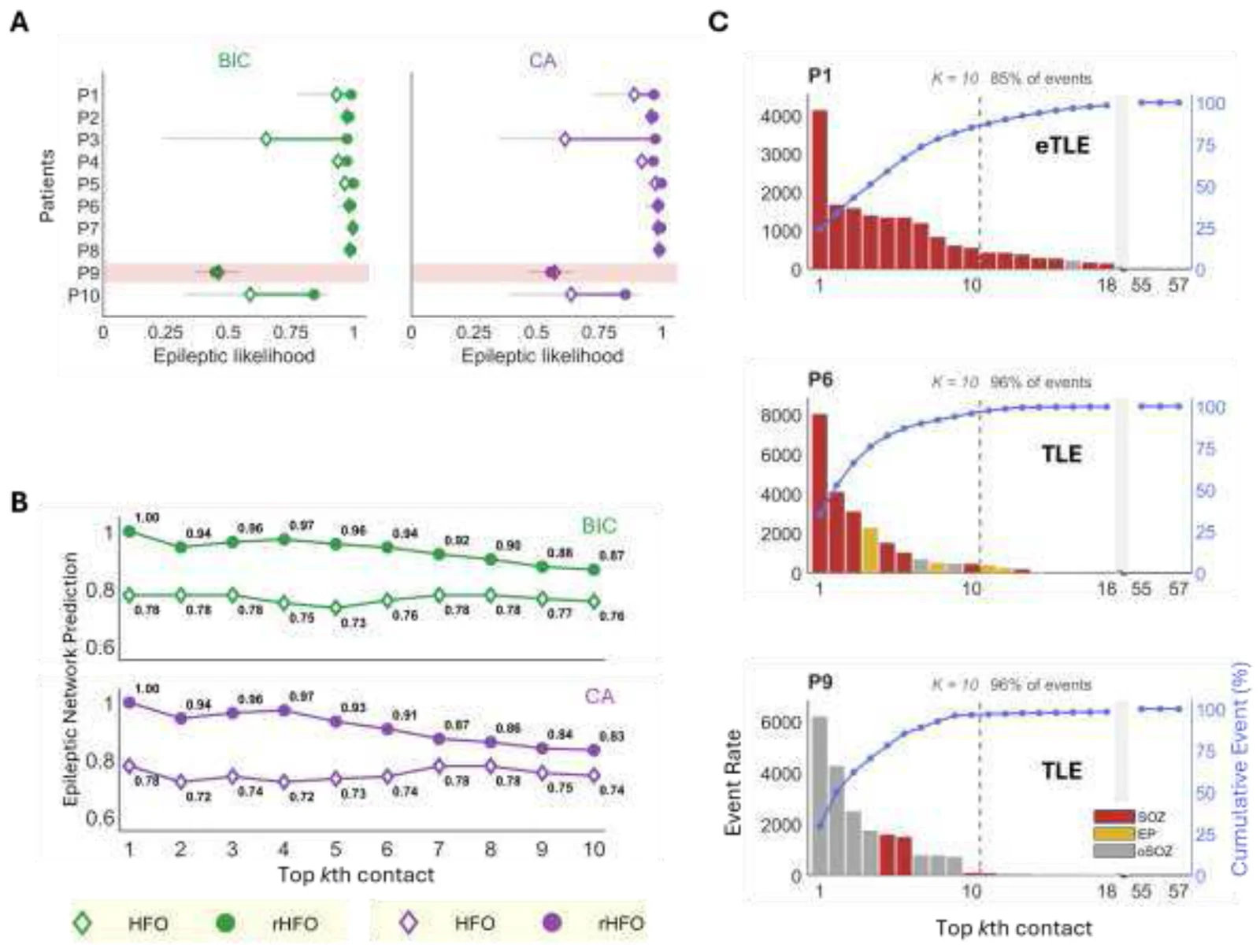
Epileptic network localization based on HFO distributions derived from BIC and CA recordings. **(A)** Subject-wise median epileptic likelihood per patient, before (open diamond) and after (filled circle) pHFO elimination, for BIC (green, left) and CA (purple, right). Gray whiskers show the interquartile range across 10-min segments over the 24-h recording. For P9, the likelihood stayed low (BIC ≈ 0.43; CA ≈ 0.54). Excluding P9, mean ± SD increased from 0.882 ± 0.167 to 0.951 ± 0.090 for BIC and from 0.887 ± 0.130 to 0.954 ± 0.068 for CA after denoising. **(B)** Channel ranking based on long-term HFO rate. Contacts were ranked by total HFO rate over 24 h, and the proportion of top channels overlapping with the clinically defined epileptic network (SOZ + early-propagation contacts) is shown for K = 1 to 10. For both systems, most of the top three and top five HFO-ranked channels overlapped with the epileptic network. Hollow diamonds: initial HFOs; filled circles: rHFOs. Green: BIC, purple: CA. **(C)** Channel ranking based on long-term HFO rate over 24 h for representative subjects (P1, P6, and P9) recorded with the BIC system. Bar plots show the total number of detected HFO events per channel, ranked from highest to lowest rate. Bars are color-coded according to anatomical category: SOZ contacts (red), early-propagation (EP) contacts (yellow), and outside-SOZ (oSOZ) contacts (gray). The dashed vertical line at K = 10 indicates the top 10 contact threshold.

Across subjects with confident SOZ localization (*n = 9*), epileptic-network likelihood increased after pHFO elimination for both recording streams (Fig. 6A). Excluding P9 (the only clinically non-SOZ localized case, which remained low in both systems: BIC ≈ 0.43; CA ≈ 0.54), the mean ± standard deviation (SD) of subject-wise epileptic likelihood increased from 0.882 ± 0.167 before pHFO elimination to 0.951 ± 0.090 afterward for BIC, and from 0.887 ± 0.130 to 0.954 ± 0.068 for CA. A linear mixed-effects analysis of segment-level likelihood confirmed a significant effect of denoising across both systems. The model intercept (BIC pre-denoising) was 0.870 ± 0.051, and the denoising effect (post vs. pre pHFO elimination) was −0.051 ± 0.006, indicating a significant increase in likelihood after pHFO elimination (*p < 0.001*). No significant main effect of recording system was observed (BIC versus CA, p = 0.14), and the recording system-by-denoising interaction was not significant (p = 0.94), indicating that the improvement after pHFO elimination was comparable between BIC and CA recordings. Random intercepts for subjects accounted for inter-individual variability (SD = 0.162); residual SD was 0.134. Beyond probabilistic epileptic-network mapping, we evaluated a clinically practical identification strategy based on channel ranking by long-term HFO rate (Fig. 6B). Across 24 h of dual-stream recordings, most of the top three and top five HFO-highest contacts overlapped with the clinically defined epileptic network. This was true for both BIC- and CA-derived HFOs, supporting the translational relevance of BIC-based HFO measurements for guiding epileptogenic-region identification. This pattern is illustrated for representative subjects (P1, P6, and P9) recorded with the BIC system, where channels ranked by total HFO count show concentration of high-rate contacts within SOZ and early-propagation regions (Fig. 6C). Here, ranking contacts by total HFO count revealed a strongly skewed distribution: a small number of channels accounted for the majority of detected events, such that the cumulative HFO proportion captured by the first few top-ranked contacts rose steeply before plateauing. This sparsity indicates that pathological HFO activity is concentrated in a limited set of contacts, allowing the principal epileptogenic channels to be localized from only the highest-rate sites rather than requiring exhaustive review of the full array. Per-subject channel rankings confirming this pattern are provided for BIC (Suppl. Fig. S6) and CA (Suppl. Fig. S7), where SOZ and EP contacts dominate the top-ranked channels in all subjects with confident localization. At the group level, coverage of the clinically defined epileptic network increased monotonically with the number of top-ranked contacts included (Suppl. Fig. S8). Both BIC and CA reached 80% coverage of SOZ regions using the top k = 10 contacts. For early-propagation regions, CA reached 80% coverage at k = 9 and BIC at k = 11.

Evaluation of SOZ classification using HFO distributions showed that, before pHFO elimination, the average sensitivity was 80.9 ± 14.1% for BIC and 82.7 ± 14.3 % for CA, with specificities of 85.0 ± 8.0% and 81.4 ± 9.8%, respectively (across 9 subjects excluding the uncertain-SOZ case P9). After pHFO elimination, sensitivity was 84.0 ± 11.1% for BIC and 82.0 ± 12.6% for CA, while specificity increased to 90.2 ± 7.6% for BIC and 90.2 ± 9.5% for CA (Suppl. Fig. S5C).

### Spectral Characteristics and Subtype-Specific HFO Detection Across BIC and CA Recordings

**Fig. 7:**
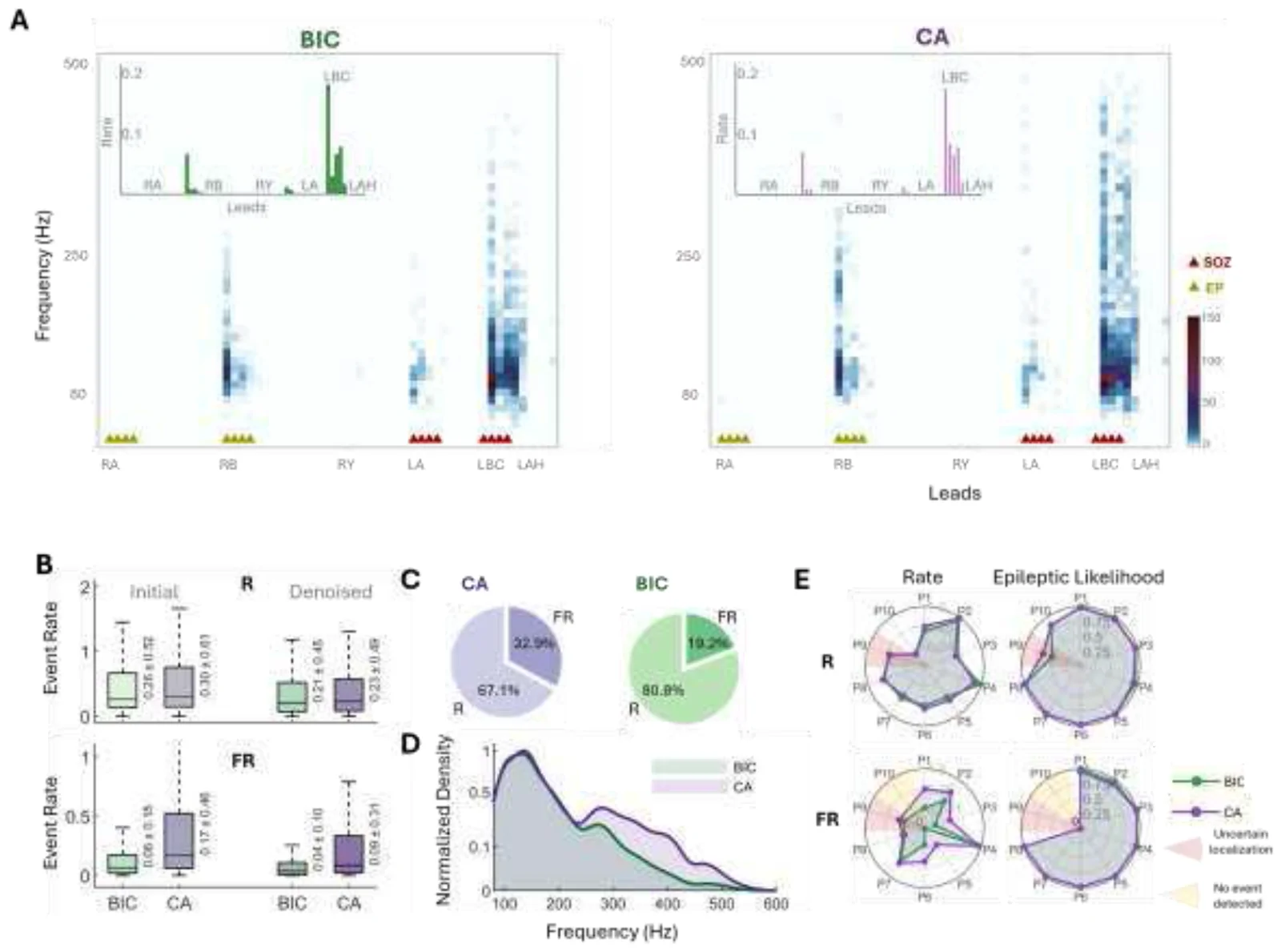
Comparison of HFO subtype rates and spatio-spectral characteristics between BIC and CA recordings. **(A)** Spatially normalized rate and spatio-spectral distribution of the highest oscillatory frequency component within detected HFO events from one representative hour of data (P6), shown for BIC (green) and CA (purple) recordings. The x-axis denotes channel index, and the y-axis indicates the maximum oscillatory frequency per event. Channels clinically annotated as SOZ and EP are indicated by red and yellow arrows, respectively. CA recordings exhibit a higher density of fast-ripple–range events, whereas corresponding BIC events are shifted toward lower oscillatory frequencies. **(B)** Boxplots of ripple (R, rR) and fast-ripple (FR, rFR) event rates across all subjects and non-overlapping 10-minute segments, shown before and after pseudo-HFO elimination for BIC and CA recordings. Overall HFO rates were comparable between systems. Ripple rates were also similar between systems, whereas fast-ripple detections were reduced relative to CA, both before and after denoising. **(C)** Proportion of ripple and fast-ripple events captured by each system across all subjects. In BIC recordings, 19.2% of detected HFOs were classified as fast ripples, compared with 32.9% in CA recordings. **(D)** Spectral frequency distribution of detected HFOs in BIC and CA recordings, demonstrating a reduced probability of fast-ripple–range frequencies in BIC and a corresponding shift toward lower-frequency ripple components. **(E)** event rate (left) and epileptic likelihood (right) computed separately for ripples and fast ripples after pHFO elimination, shown as radial plots across subjects (P1 to P10 arranged clockwise from the top) derived from the full 24-h recording. BIC-derived measures are shown in green and CA-derived measures in purple. Ripple-based analyses showed consistently elevated rates and epileptic likelihood across most subjects with confident localization in both systems. Fast-ripple–based epileptic likelihood reached zero in the subject with uncertain-localization. P10 was excluded from the fast-ripple analysis due to insufficient event counts.

When HFO subtypes were analyzed separately, systematic differences between R and FR events were observed across the two recording systems. To characterize these effects at the signal level, the maximum oscillatory frequency within each detected event was estimated using an adaptive sparse regenerative model^32^. Two-dimensional spatio-spectral histograms were constructed for one hour of data from a representative subject (P6), with channel index on the x-axis and the highest oscillatory frequency per event on the y-axis. As shown in Fig. 7A, CA recordings exhibited a higher density of events dominated in FR-range frequencies, whereas corresponding events in the BIC stream clustered predominantly below 200 Hz, reflecting a shift toward lower oscillatory components.

For R events, detection rates were similar between BIC and CA, both before denoising (R: BIC 0.26 ± 0.52, CA 0.30 ± 0.61; *p = 1.000*) and after pHFO elimination (rR: BIC 0.21 ± 0.45, CA 0.23 ± 0.49; *p = 0.695*), indicating no statistically significant differences. In contrast, FR detections were significantly lower in BIC recordings compared with CA. Initial FR rates were 0.06 ± 0.15 for BIC versus 0.17 ± 0.46 for CA (*p = 0.002*), and post-denoising rFR rates were 0.04 ± 0.10 and 0.09 ± 0.31, respectively (*p = 0.002*). Across the dataset, FR detections in BIC accounted for approximately 44% of those observed in CA, reflecting a substantial and statistically significant reduction in FR detection (Fig. 7B).

The overall proportion of R and FR events captured across all subjects is shown in Fig. 7C. In BIC recordings, 19.2% of detected HFOs were classified as FRs, compared with 32.9% in CA recordings, further illustrating the relative under-detection of high-frequency components in the implantable system. These subtype-specific differences are primarily attributable to the reduced effective spectral bandwidth of BIC, which attenuates higher-frequency oscillations characteristic of FRs. As shown in Fig. 7D, the spectral frequency distribution of detected HFOs reveals a clear shift toward lower dominant frequencies in BIC, with a reduced probability of events extending into the fast-ripple range. Elevated background noise above ~200 Hz in the BIC stream further limits reliable FR detection, causing some events to fall below detection thresholds. The spatial distribution of R and FR events across channels was quantified using the Gini coefficient, reflecting the sparsity of events across recording sites. In BIC recordings, median Gini coefficients were 0.869 (0.053) for R and 0.904 (0.047) for FR, indicating a higher sparsity of FRs across fewer channels. In CA recordings, median Gini coefficients were 0.866 (0.060) for R and 0.891 (0.052) for FR, showing a similar trend. Wilcoxon signed-rank tests revealed a highly significant difference between Rs and FRs in both BIC and CA (*p < 0.001*), confirming that Rs are more widely distributed than FRs across recording channels.

Finally, both event rate and epileptic likelihood were computed separately for R and FR subgroups across all subjects (Fig. 7E). Subject-level values were derived as medians across non-overlapping 10-min segments over the full 24-h recording and visualized as radial plots with BIC in green and CA in purple. Ripples were consistently more abundant than fast ripples across subjects in both recording streams, with FR event rates substantially lower and, in several subjects, sparse or near absent. For R events, both BIC and CA streams showed robust rates and reliable localization to the clinically defined epileptic network. For FRs, the markedly lower event density was accompanied by reduced or absent epileptic likelihood in several subjects, including a likelihood of zero for P9, the subject with an inconclusive SOZ. P10 was excluded from the FR analysis because of insufficient event counts.

## Discussion

This study demonstrates that implant-grade neural sensing coupled with artifact-resilient computational intelligence can preserve clinically meaningful spatial HFO biomarkers of epilepsy. Using a benchtop implementation of the Brain Interchange (BIC) system during simultaneous bedside iEEG recordings, we evaluated whether the sensing constraints expected for a chronic wireless implantable neural interface preserve the information required to identify epileptogenic tissue. Rather than simply benchmarking a recording device against a clinical amplifier, this work establishes a translational framework for transferring HFO biomarkers from hospital-based monitoring toward chronic implantable neuromodulation.

Despite lower sampling rate, reduced analog bandwidth, and an elevated high-frequency noise floor, implant-derived HFOs retained strong spatial correspondence with clinical recordings and localized epileptogenic tissue with comparable performance following artifact rejection. These findings suggest that preserving the spatial organization of pathological HFO activity, rather than reproducing every individual high-frequency event, may represent the critical requirement for future biomarker guided monitoring and adaptive neuromodulation.

### Impact of Hardware Constraints on HFO Sensing

The BIC system operates under substantially tighter engineering constraints than a tethered clinical amplifier, including lower sampling rate (1 kHz), limited analog bandwidth (325 Hz), low power consumption (implant ≤ 400 mW, and total benchtop unit ~ 1W), and restricted thermal dissipation. These constraints increased the high-frequency noise floor and reduced sensitivity to fast ripples, yet they did not substantially alter the spatial distribution of clinically informative HFO activity.

The elevated HFO-band background activity is unlikely to be explained by wireless data transmission alone because packet loss remained below 1% throughout the recordings, a level previously shown^33^ to have minimal impact on HFO detection. Nevertheless, these controlled EMU recordings almost certainly underestimate the communication challenges expected during chronic ambulatory use, where patient mobility, posture changes, environmental interference, and prolonged wireless transmission are likely to increase both packet loss and artifact burden. Consequently, the present packet-loss analysis should be interpreted as demonstrating stable communication under controlled bedside conditions rather than predicting long-term ambulatory performance.

Differences in digitization are similarly unlikely to explain the increased noise floor because quantization noise is negligible relative to the analog front-end noise of both systems. Instead, the observed increase in high-frequency background activity is more consistent with expected characteristics of implant-grade recording electronics, including elevated input-referred thermal noise associated with low-power analog amplifiers and potential limitations of the front-end application-specific integrated circuit (ASIC) architecture^34-36^. Miniaturized implantable neural recording systems face fundamental tradeoffs between power consumption, physical size, and noise performance^37^. Because the benchtop BIC system shares the implant's analog front end electronics ^38^, these recordings provide a realistic assessment of the sensing constraints expected for the chronic implantable platform. Low power analog front ends operate with reduced bias currents and limited supply headroom, which increases the contribution of thermal noise at the input stage^39^. This behavior is well characterized in the neural amplifier design literature through metrics such as the noise efficiency factor, which quantifies the relationship between input-referred noise and power consumption; achieving noise levels comparable to clinical-grade systems would require power budgets and circuit area that are incompatible with chronic implantable form factors^40-43^. Furthermore, because implanted electronics must limit tissue heating, chronic systems impose strict constraints on total and per-channel power dissipation budgets^44-46^.

The impact of these hardware constraints was frequency dependent. Ripple-band activity (80–200 Hz), which lies well within the effective recording bandwidth and typically exhibits larger peak-to-peak amplitudes, was largely preserved in BIC recordings, whereas FR detection was reduced relative to the clinical amplifier, with approximately 44% preservation. These findings indicate that implantable architecture did not produce a uniform loss of HFO information, but instead selectively reduced sensitivity to the highest-frequency events, consistent with prior reports that FRs generally have smaller amplitudes and lower signal-to-noise ratios than ripples^27,47,48^ and their consequent vulnerability to limited analog bandwidth, lower sampling rate, and elevated high-frequency front-end noise^34,35,42,44^.

### Artifact-Resilient Computational Analysis as an Integral Component of Implantable HFO Sensing

Hardware–computational intelligence integration is particularly important for prolonged recordings, where muscle activity, movement, electromagnetic interference and environmental noise can generate high-frequency transients that overlap spectrally with pathological HFOs^31,49,50^. A central finding of this study is that computational analysis is not merely a post-processing step but an integral component of implantable biomarker sensing. Approximately one-quarter of all candidate events detected by both recording systems were classified as pseudo-HFOs, demonstrating that artifact contamination remains a substantial challenge during prolonged intracranial monitoring regardless of acquisition platform.

Artifact rejection substantially improved localization performance for both systems and largely eliminated differences between the implantable interface and the clinical amplifier. Rather than compensating for hardware deficiencies, the computational framework maximized extraction of clinically meaningful information by suppressing physiological and environmental artifacts that would otherwise obscure pathological HFO distributions. These findings therefore demonstrate that successful translation of HFO biomarkers depends on joint optimization of implant-grade sensing and artifact-resilient computation rather than recording hardware alone.

### Spatial Biomarker Fidelity

The most important translational observation was that incomplete recovery of individual HFO events did not produce a proportional loss of clinically relevant spatial information. Although fast-ripple detection was reduced, pathological HFO activity remained concentrated within the same anatomically informative channels. We refer to this preservation of anatomical distribution and relative channel ranking as spatial biomarker fidelity.

Representative time-matched events in Fig. 2B suggest that some ripple–fast-ripple complexes (RwFRs) detected by the clinical amplifier retain a detectable ripple-band component in the BIC recordings, whereas their fast-ripple components are attenuated or obscured by the elevated high-frequency background. Consequently, subtype classification may differ between recording systems while the spatial localization of pathological activity remained largely preserved. Consistent with this interpretation, ripple distributions demonstrated excellent agreement between systems, whereas fast-ripple distributions exhibited greater variability because of their lower amplitude and increased sensitivity to hardware constraints.

The subject with uncertain clinical localization (P9) illustrates the potential value of this approach. HFOs localized outside the clinically defined seizure-onset zone in both recording systems, suggesting that the discrepancy reflected uncertainty in the underlying epileptogenic network rather than failure of the implantable device. Chronic implantable monitoring may therefore provide complementary information beyond conventional EMU evaluation by repeatedly characterizing spatial HFO dynamics over extended periods. Such longitudinal measurements may identify persistent pathological hubs or alternative epileptogenic regions that are difficult to recognize during limited presurgical recordings.

These observations suggest that chronically implantable systems need not recover every individual HFO event to provide clinically meaningful information. Instead, preserving stable or evolving spatial patterns of pathological activity may represent a more important objective for long-term biomarker sensing.

### Implications for Adaptive Neuromodulation

The present findings support the potential for a shift from viewing HFOs as static biomarkers of seizure-onset localization toward dynamic biomarkers capable of tracking evolving epileptogenic networks. Implantable neural interfaces capable of prolonged sensing may therefore provide information unavailable during conventional EMU evaluation, enabling adaptive stimulation strategies based on longitudinal changes in pathological network activity rather than a single presurgical recording session. By demonstrating that HFO distributions retain spatial specificity over 24-hour recordings and remain robust after artifact elimination, this work supports the feasibility of incorporating HFO-based logic into future adaptive stimulation paradigms^51^.

These results are particularly relevant given the rapid emergence of bidirectional sensing-enabled neuromodulation platforms^14,23,24,38,52-56^. Together, current and next-generation implantable systems create an opportunity to integrate chronic biomarker sensing with adaptive stimulation across epilepsy and other neurological disorders^7,8,10,12,57,58^. Our findings indicate that implantable systems may not require event-level performance equivalent to clinical bedside amplifiers to provide clinically useful biomarker information. Instead, preserving stable spatial representations of pathological neural activity over extended timescales may represent a more practical and clinically relevant design objective for future closed-loop neuromodulation systems.

### Limitations and Future Directions

Several limitations should be considered. First, recordings were obtained using benchtop versions of the implantable system during prolonged EMU monitoring rather than fully implanted ambulatory use. Although the benchtop and implantable platforms share the same analog front-end electronics, acquisition architecture, and wireless communication framework, the controlled bedside environment likely underestimates motion-related artifacts, environmental interference, and packet loss encountered during everyday life. Consequently, both HFO detection performance and wireless communication robustness should be validated during chronic ambulatory deployment.

Second, reduced fast-ripple sensitivity represents an expected consequence of the current implant architecture, reflecting the combined effects of analog bandwidth, sampling rate, and front-end noise. Future implant generations incorporating broader bandwidth, lower-noise analog front ends, improved ASIC design, and higher sampling rates may further enhance detection of high-frequency biomarkers while preserving the power efficiency required for chronic implantation. Beyond hardware improvements, adaptive sensing strategies may provide an additional opportunity to improve high-frequency biomarker recovery. Rather than continuously sampling all available channels (e.g., 32 channels) at a uniform sampling rate, future implantable systems could dynamically allocate acquisition bandwidth by identifying HFO-rich regions during chronic monitoring and selectively increasing the sampling rate or bandwidth for those channels. Such adaptive sampling strategies could improve fast-ripple recovery while maintaining overall power and telemetry constraints.

The HFO detection and pHFO classification framework presented here currently relies on external processing rather than on-implant computation. This offers an advantage at this stage, since processing can be performed on an external device with a larger power budget and richer computational resources than the implant itself can support. A future direction is to develop low-complexity detectors that operate within the implant's restricted power and computational budget, allowing this framework to eventually run directly on implantable hardware. More broadly, future studies should determine whether the long-term spatial stability of HFO biomarkers, rather than complete recovery of individual HFO events, provides the most reliable physiological substrate for adaptive neuromodulation during chronic ambulatory recordings. Together, adaptive sensing, artifact-resilient computation, and longitudinal network monitoring may enable implantable neural interfaces to dynamically track evolving epileptogenic networks and guide personalized closed-loop stimulation strategies.

## Conclusions

In summary, this study demonstrates that clinically meaningful HFO biomarkers can be translated from conventional hospital-based recordings to an implant-grade neural sensing architecture despite reduced sampling rate, limited analog bandwidth, and an elevated high-frequency noise floor. All recordings were obtained using the benchtop implementation of the Brain Interchange (BIC) system. Because the benchtop and implantable platforms share the same analog front-end electronics, acquisition architecture, sampling rate, and signal-conditioning pipeline, these findings provide a realistic assessment of the sensing characteristics expected from the implantable device, although prospective validation during chronic ambulatory use remains necessary. By integrating hardware-aware signal acquisition with artifact-resilient computational analysis, implant-derived HFOs preserved spatial biomarker fidelity and achieved epileptogenic tissue localization comparable to a clinical recording system. Collectively, these findings establish a translational framework for moving HFO biomarkers from the epilepsy monitoring unit to chronic implantable neural interfaces and provide a foundation for future biomarker-guided adaptive neuromodulation in epilepsy.

## Methods

### Data Acquisition and Study Design

Intracranial electrophysiological data were collected from 10 patients with drug-resistant epilepsy who underwent invasive iEEG monitoring as part of their presurgical evaluation in the EMU at Mayo Clinic in Rochester, Minnesota. All study procedures were approved by the Mayo Clinic Institutional Review Board (IRB no. 23-008859), and the study was registered at ClinicalTrials.gov (NCT05439655). All experiments were conducted in accordance with relevant guidelines and regulations. Written informed consent was obtained from all participants or their legally authorized representatives prior to inclusion in the study.

Neural recordings were acquired using a benchtop version of the Brain Interchange System (BIC, CorTec GmbH, Freiburg, Germany), an externally powered, bidirectional neural interface supporting 32-channel sensing and stimulation, wireless data transmission, and continuous broadband recording at a sampling frequency of 1 kHz. In our earlier work^38^, the BIC system was evaluated for its ability to capture interictal spikes and lower-frequency (<80 Hz) neural biomarkers during intracranial monitoring using a rapid prototyping framework based on MATLAB and Simulink. Building on this framework, the present study focuses on the detection and characterization of HFOs using the CorTec BIC.

Continuous iEEG recordings were obtained using two synchronized BIC benchtop units per subject during the final 2 to 3 days of prolonged EMU monitoring. Each unit provided 32 recording channels, resulting in up to 64 BIC channels per patient. Electrode contacts were selected to provide broad spatial coverage across regions with higher and lower clinical likelihoods of seizure-onset involvement based on prior noninvasive studies, clinical hypotheses, and observations during EMU monitoring. Signals from the selected contacts were split into two parallel acquisition streams, with one routed to the BIC systems and the other to the standard-of-care Natus Quantum clinical amplifier (CA; Natus Medical Inc., Middleton, WI, USA). BIC recordings were sampled at approximately 1 kHz, whereas CA recordings were acquired at 2 kHz. Both streams were recorded simultaneously for approximately 24 hours to enable direct comparison of signal characteristics and HFO detection performance.

The two BIC units were synchronized using a shared external clock source^59^. Each BIC system used independent ground and reference contacts selected from electrodes located in white matter to minimize physiological interference and improve recording stability. In contrast, the CA used standard clinical grounding and referencing according to routine EMU practice.

BIC data were acquired using benchtop investigational units without chronic implantation of the device hardware. Power was delivered externally through inductive coupling using a magnetic coil, and neural data were transmitted wirelessly to the communication unit and subsequently streamed to a host computer through USB. Data acquisition and system control were implemented using a custom MATLAB/Simulink-based interface developed with the CorTec application programming interface. Additional details regarding the system architecture, data buffering, and communication protocols have been described previously^38^.

Temporal alignment between the combined dual-BIC recordings and corresponding CA data was performed offline using multichannel convolution-based template matching, as described below. Following synchronization, recordings from both systems were analyzed using the same HFO detection, pHFO elimination, and spatial-mapping framework. Clinical annotations, including SOZ contacts, were provided by the clinical team based on routine presurgical evaluations. Cohort characteristics are summarized in Table 1.

### Data Synchronization

All recorded iEEG data were processed offline prior to HFO analysis. BIC recordings were acquired at approximately 1 kHz with actual sampling frequencies of the two units exhibiting small non-integer deviations (e.g., ~995–1005 Hz) due to independent clocking characteristics. As a result, long-duration recordings exhibited gradual temporal drift relative to the CA and, if uncorrected, between the two BIC units themselves. To address this, a two-stage temporal alignment strategy was implemented.

In the first stage, recordings from the BIC units were synchronized using a shared external master clock incorporated into the acquisition framework, as described in our previously published work (CNELBehv ^60^). This ensured consistent sample-wise alignment between the BIC data streams throughout prolonged recording and enabled their concatenation into a unified 64-channel BIC dataset (Fig. S1).

In the second stage, temporal alignment between the combined dual-BIC data and the corresponding clinical recordings was performed to support device-level comparisons. Given the gradual clock drift over extended durations, alignment was performed independently within non-overlapping 10-minute segments across the 24-hour recording. Within each segment, short reference templates (5 seconds) extracted from the clinical data were used to identify the corresponding time windows in the BIC data using multichannel convolution-based template matching. This process compensated for residual timing offsets and ensured accurate correspondence between BIC and clinical recordings at the sample level. Following alignment, each subject’s dataset consisted of 144 temporally matched 10-minute segments of dual-BIC (64 channels, 1 kHz) and corresponding clinical (64 channels, 2 kHz) data. After synchronization, iEEG signals from both the BIC and the clinical system were processed using the same signal processing and HFO detection pipeline, allowing direct comparison of HFO characteristics across systems. The schematic overview of the simultaneous BIC/CA recording configuration and the post hoc synchronization pipeline is provided in Suppl. Fig. S1.

### HFO Detection and Artifact Rejection

Synchronized iEEG signals were re-referenced using bipolar derivations to remove common-mode activity shared between neighboring electrode contacts. Bipolar signals were then band-pass filtered between 80 and 500 Hz using a 64-tap finite impulse response (FIR) filter implemented with a zero-phase forward–backward filtering procedure, ensuring that no phase delay was introduced into the filtered waveforms. An identical filter design was used for both recording systems, with filtering performed at each signal’s native sampling rate (BIC: ~1 kHz; CA: 2 kHz), thereby preserving the same absolute frequency range while considering system-specific Nyquist limits.

HFO detection was performed using a two-stage framework consisting of a deterministic amplitude-based candidate detector followed by a machine-learning-based artifact rejection stage (Fig. 1B). The initial detector was intentionally configured to maximize sensitivity by identifying a broad pool of candidate oscillatory events, which were subsequently refined through artifact classification. Initial HFO candidates were identified using a single-band, dual-threshold amplitude detector applied to the 80–500 Hz filtered signal. To trigger the detection process, candidate events were required to exceed an upper detection threshold at least once and to exhibit sustained oscillatory activity above a lower threshold, with a minimum of three oscillatory cycles. Threshold levels were defined as multiples of the estimated background noise standard deviation (σ), using multipliers m = [3.5, 4.5] for the lower and higher thresholds, respectively^61^. To further suppress isolated sharp transients, candidate events were required to satisfy a semi-symmetric oscillatory criterion in which threshold crossings of opposite polarity were approximately balanced within a predefined tolerance. This constraint favors sustained oscillatory activity while avoiding unnecessarily strict waveform symmetry. In addition, a minimum absolute amplitude threshold was applied to both recording systems. These lower bound rejects candidate events with amplitudes too small to reflect genuine neural activity, particularly in regions where amplifier saturation or recovery produces near-flat baseline segments with a standard deviation approaching zero, which can cause the adaptive threshold to fall to artificially low levels and admit non-neural detections. Identical detection parameters were applied to both recording systems. To improve specificity, all detected events were subsequently processed using a previously validated pHFO elimination pipeline^32^. This framework employs a sparse signal representation strategy to distinguish true neural HFOs from artifact-related events based on their waveform morphology rather than conventional time-frequency features. Each candidate event is represented in a sparse fashion using orthogonal matching pursuit (OMP) with a fixed, analytically designed redundant Gabor dictionary composed of oscillatory atoms spanning the HFO frequency range. The sparse representation was characterized using complementary global and local measures, including the approximation error, a variation factor describing residual sharp transients, and the number of selected atoms at the power-line frequency to identify line-noise contamination. These features, concatenated across successive iterations of the sparse approximation, are provided as input to a previously trained random forest classifier that assigns each candidate event to either the true-HFO or pseudo-HFO class. The classifier was identical to that described previously^32^, where it was trained and validated using approximately 6,400 manually labeled true-HFO and pseudo-HFO events from 16 independent patients. The pretrained classifier was applied without retraining to all candidate events detected in the present study. No labels from the current dataset were used during classifier development, enabling independent evaluation of the computational framework without dataset-specific optimization or information leakage. Because the classifier was developed independently of the present dataset, its performance reflects generalization to a new recording platform rather than optimization for the current study.

This classification step removed non-neural high-frequency transients prior to subsequent spatial and statistical analyses. All preprocessing, HFO detection, feature extraction, and pseudo-HFO classification steps were identical for the implantable and clinical recording systems unless otherwise specified. This two-stage framework separates sensitive candidate detection from artifact classification, enabling robust identification of pathological HFOs while minimizing contamination from physiological and environmental high-frequency transients during prolonged intracranial recordings. Consequently, differences observed between recording systems primarily reflect acquisition hardware rather than differences in signal processing or classifier optimization.

### Performance Metrics for Comparing BIC and Clinical iEEG HFO Analyses

To quantitatively compare HFO detection and spatial localization performance between the implantable BIC system and the CA, multiple complementary signal- and biomarker-level metrics were evaluated:

#### 1. Noise characterization

Background noise differences were assessed using power spectral density (PSD) estimates across all non-corrupted channels for each system. PSDs were aggregated to characterize broadband and HFO-band (80–500 Hz) noise profiles. In addition, RMS amplitudes were computed within the HFO band over non-overlapping 10-minute segments across all channels and subjects. PSD and RMS distributions were used to quantify relative noise floors between BIC and CA recordings in HFO band (80-500 Hz).

#### 2. HFO rate and subtype comparisons

Normalized rates of total HFOs, Rs, and FRs were computed after pHFO elimination. Rate distributions were compared across subjects within clinically defined SOZ and EP channels, which were treated as the primary epileptogenic regions.

#### 3. Spatial alignment of detections across systems

For each subject, HFO rates across channels were represented as vectors, with each element corresponding to the normalized HFO rate of an individual channel, computed over 10-minute segments across 24 hours of recording. Cosine similarity was used to quantify agreement in the spatial pattern of HFO activity between systems, independent of absolute rate magnitude:

(1) $$\cos(\theta )=\frac{\left(x.y\right)}{\left(\|x\|.\|y\|\right)}$$

where x and y denote the vectors of normalized channel HFO rates for the BIC and CA systems, respectively. Analyses were performed before and after pHFO elimination to assess the effect of artifact removal across-systems.

#### 4. SOZ likelihood and channel ranking

SOZ likelihood was defined as a rate-normalized measure comparing HFO activity inside, outside the SOZ and EP contacts. Let $r_{i}^{k}$ denote the normalized HFO rate of channel i in segment k, and let SOZ, EP, and nSOZ denote the corresponding channel sets for a given subject, with $N_{SOZ}$, $N_{EP}$, and $N_{oSOZ}$ their respective channel counts. For each non-overlapping 10-minute segment k, the mean HFO rate was computed separately for SOZ and non-SOZ contacts:

(2) $$R_{SOZ}^{k}=\frac{1}{N_{SOZ}}\sum_{i\in SOZ}r_{i}^{k}$$

(3) $$R_{EP}^{k}=\frac{1}{N_{EP}}\sum_{i\in EP}r_{i}^{k}$$

(4) $$R_{OSOZ}^{k}=\frac{1}{N_{oSOZ}}\sum_{i\in oSOZ}r_{i}^{k}$$

and SOZ likelihood was calculated as their ratio:

(5) $$L_{SOZ}^{k}=\frac{R_{SOZ}^{k}}{R_{SOZ}^{k}+R_{nSOZ}^{k}+R_{EP}^{k}}$$

yielding a bounded metric between 0 and 1. Segment-level values were averaged across the full 24-hour recording to obtain a subject-level SOZ likelihood.

Epileptogenic likelihood was defined analogously but extended to include EP contacts. Mean HFO rates were computed separately for SOZ, EP, and oSOZ contacts, and Epileptogenic likelihood was calculated as the combined SOZ and EP rate divided by the sum of SOZ, EP, and non-epileptogenic rates.


(6) $$L_{EZ}^{k}=\frac{R_{SOZ}^{k}+R_{EP}^{k}}{R_{SOZ}^{k}+R_{nSOZ}^{k}+R_{EP}^{k}}$$

In addition, channels were ranked by HFO rate within each subject, and the fraction of the top-k ranking channels overlapping with clinically defined SOZ contacts (k = 10) was quantified to assess prioritization performance.


(7) $$F_{overlap}^{k}=\frac{|top_{k}\cap SOZ|}{k},if\;N_{SOZ}\geq k$$

#### 5. SOZ classification performance metrics

Finally, sensitivity and specificity for SOZ detection were computed for both recording systems using established statistical definitions^62^. Following spatial normalization of HFO rate distributions, channels were classified as SOZ-positive or SOZ-negative based on predefined biomarker thresholds. True positives (TP) were defined as clinically identified SOZ channels correctly classified by elevated HFO activity; false positives (FP) as non-SOZ channels incorrectly classified as SOZ; true negatives (TN) as non-SOZ channels correctly rejected; and false negatives (FN) as SOZ channels not identified by the biomarker distribution. Sensitivity and specificity were then calculated using standard formulations^63^.


(8) $$Sensitivity=\frac{TP}{TP+FN}$$

(9) $$specificty=\frac{TN}{TN+FP}$$

These metrics were evaluated separately for initial HFO detections and after pHFO elimination, for both BIC and CA recordings.

#### 6. Spatial Sparsity of HFO Distributions

Spatial sparsity of channel-wise HFO distributions was quantified using the Gini coefficient,

(10) $$G=\frac{\sum_{i=1}^{n}\sum_{j=1}^{n}|x_{i}-x_{j}|}{2n\sum_{i=1}^{n}x_{i}}$$

where $x_{i}$ denotes the normalized HFO rate of channel i and n is the number of recording channels. Values approaching zero indicate uniformly distributed HFO activity across recording sites, whereas values approaching one indicate that HFO activity is concentrated within a small subset of channels. Because pathological HFOs are expected to cluster within epileptogenic tissue, higher Gini values indicate greater spatial localization of biomarker activity. We selected the Gini coefficient because our objective was to quantify spatial concentration of pathological activity rather than distributional uncertainty. Unlike entropy, which measures uncertainty and depends on both the number and relative occupancy of active channels, the Gini coefficient directly quantifies inequality in channel-wise HFO distributions and provides an intuitive measure of spatial sparsity that is independent of the absolute number of detected events.

### Statistical Analysis

Data are reported as mean ± standard deviation (SD) across subjects. Group comparisons between BIC and CA recordings were performed using the Wilcoxon signed-rank test for paired data at the subject level. To quantify the magnitude and direction of differences, the rank-biserial correlation was calculated as an effect size for all comparisons. To assess segment-level variability and simultaneously evaluate effects of device type and pHFO elimination, a linear mixed-effects model (LME) was applied with segment-level SOZ likelihood as the dependent variable and a random intercept per subject. Statistical significance was set at *p < 0.05*. Finally, Cliff’s delta (δ) was computed at the subject level as a nonparametric effect size to quantify the dominance of background noise RMS values in BIC relative to CA recordings. Group-level medians and 95% confidence intervals were estimated via bootstrap resampling, and significance was assessed using a Wilcoxon signed-rank test against zero.

## Supplementary Material

This is a list of supplementary files associated with this preprint. Click to download.

• SupplMaterial.docx

## Figures and Tables

**Table 1-:** Patient presurgical hypotheses and clinical Info

Patient	SOZ Regions	Early Propagation Regions	Epilepsy Type	Primary Hypothesis	MRI Findings	Other Information
**P1**	Superior frontal gyrus; medial superior frontal gyrus; inferior frontal gyrus	N/A	eTLE	Bifrontal onset	Left frontal encephalomalacia	MEG: bifrontal dipoles, some with left-leading activity
**P2**	Middle temporal gyrus / posterior operculum	Bilateral amygdala and hippocampus	TLE	Right temporoparietal	Right temporal and insular abnormalities	SPECT: right temporoparietal
**P3**	Right amygdala/hippocampus; left hippocampus	N/A	TLE	Right temporal–insular	Non-lesional	ESI: insular
**P4**	Inferior lesion	N/A	eTLE	Right parietal FCD	FCD at postcentral sulcus	SPECT: right parietal
**P5**	Inferior lesion; anterior cingulate	Gyrus rectus; inferior frontal gyrus	eTLE	Right frontal lobe epilepsy	Right parasagittal frontal probable FCD	N/A
**P6**	Left hippocampus and amygdala	Right hippocampus and amygdala	TLE	Hypothalamic hamartoma, limbic	Hypothalamic hamartoma	N/A
**P7**	Left amygdala; left hippocampus; lingual gyrus; para hippocampal gyrus	Inferior mesial temporal pole	TLE	Left limbic	No Imaging	PET: left mesial temporal; ESI: left medial posterior temporal
**P8**	Mid–superior insula; piriform cortex; mesial temporal pole	Right amygdala and hippocampus	TLE	Right > left limbic	No Imaging	PET: bitemporal; SPECT: left parieto-occipital
**P9**	Supramarginal gyrus; inferior temporal pole; inferior mesial temporal pole	Lateral lesion	eTLE	Left posterior temporal	Left parietal encephalomalacia	MEG: left temporal; PET: left temporal hypometabolism
**P10**	Left amygdala and hippocampus	N/A	TLE	Left temporal	MRI negative	MEG: left temporal (medial & lateral); PET: left temporal (L > R)

TLE, temporal lobe epilepsy; eTLE, extratemporal lobe epilepsy; FCD, focal cortical dysplasia; MRI, magnetic resonance imaging; MEG, magnetoencephalography; PET, positron emission tomography; SPECT, single-photon emission computed tomography; ESI, electrical source imaging; N/A, not available.

## Data Availability

All electrophysiological data used in this study, including raw iEEG recordings acquired simultaneously with the benchtop BIC and the corresponding clinical amplifier, are available through the Data Archive for the BRAIN Initiative (DABI) under accession Questions regarding the dataset may be directed to the corresponding author at ince.nuri@mayo.edu

## Code availability

All custom code used to generate the results reported in this study, including data preprocessing, cross-system synchronization, amplitude-based HFO candidate detection, pseudo-HFO elimination, and the associated classification model, is available through the Ince Laboratory GitHub repository at https://github.com/InceLab.
